# High-Throughput Cell Concentration Using A Piezoelectric Pump in Closed-Loop Viscoelastic Microfluidics

**DOI:** 10.3390/mi12060677

**Published:** 2021-06-09

**Authors:** Jeeyong Kim, Hyunjung Lim, Hyunseul Jee, Seunghee Choo, Minji Yang, Sungha Park, Kyounghwa Lee, Hyoungsook Park, Chaeseung Lim, Jeonghun Nam

**Affiliations:** 1 Department of Laboratory Medicine, College of Medicine, Korea University, Seoul 08307, Korea; emperorjy@naver.com; 2 Department of Obstetrics and Gynecology, College of Medicine, Korea University, Seoul 08307, Korea; hyunjunglim.email@gmail.com; 3 Artificial Intelligence(AI)-Bio Research Center, Incheon Jaeneung University, Incheon 21987, Korea; jhs603@korea.ac.kr (H.J.); tmdgl2887@gmail.com (S.C.); chymj12172947@gmail.com (M.Y.); m000855@jeiu.ac.kr (S.P.); lee12042@jeiu.ac.kr (K.L.); muni24@jeiu.ac.kr (H.P.); 4 Department of Medical Sciences, Graduate School of Medicine, Korea University, Seoul 08307, Korea; 5 Department of Song-do Bio Engineering, Incheon Jaeneung University, Incheon 21987, Korea

**Keywords:** closed-loop, concentration, piezoelectric pump, viscoelastic fluid, high-throughput

## Abstract

Cell concentration is a critical process in biological assays and clinical diagnostics for the pre-treatment of extremely rare disease-related cells. The conventional technique for sample preconcentration and centrifugation has the limitations of a batch process requiring expensive and large equipment. Therefore, a high-throughput continuous cell concentration technique needs to be developed. However, in single-pass operation, the required concentration ratio is hard to achieve. In this study, we propose a closed-loop continuous cell concentration system using a viscoelastic non-Newtonian fluid. For miniaturized and integrated systems, two piezoelectric pumps were adopted. The pumping capability generated by a piezoelectric pump in a microfluidic channel was evaluated depending on the applied voltage, frequency, sample viscosity, and channel length. The concentration performance of the device was evaluated using 13 μm particles and white blood cells (WBCs) with different channel lengths and voltages. In the closed-loop system, the focused cells collected at the center outlet were sent back to the inlet, while the buffer solution was removed to the side outlets. Finally, to expand the clinical applicability of our closed-loop system, WBCs in lysed blood samples with 70% hematocrit and prostate cancer cells in urine samples were used. Using the closed-loop system, WBCs were concentrated by ~63.4 ± 0.8-fold within 20 min to a final volume of 160 μL using 10 mL of lysed blood sample with 70% hematocrit (~3 cP). In addition, prostate cancer cells in 10 mL urine samples were concentrated by ~64.1-fold within ~11 min due to low viscosity (~1 cP).

## 1. Introduction

In biological assays and clinical diagnostics, sample preconcentration is a critical process for the pre-treatment of extremely rare disease-related cells to improve detection sensitivity [1]. Conventionally, centrifugation is the most widely used method to enrich cells into smaller volumes. However, centrifugation has several limitations. First, the centrifugation process is a batch operation, requiring expensive and bulky apparatuses, and which is inadequate for use at the point of care. Second, cells can be physically damaged due to the mechanical shear force exerted on the cells during centrifugation [2,3]. Next, a large volume reduction requires multiple centrifugation steps in a series. In particular, in rare cell applications (for example, circulating tumor cells, fungus, and fetal cells in maternal blood), the final resuspension volume can be a microliter-scale volume to achieve an appropriate cell concentration [1]. However, during multiple steps, manual transfer steps are required, and sample loss can occur.

To address the above-mentioned limitations of the centrifugation process for cell concentration, microfluidic tools for cell concentration have been developed using dielectrophoretic [4,5], acoustic [6,7,8], gravitational [1], and hydrodynamic forces [9]. Recently, viscoelastic non-Newtonian microfluidics have gained much attention, owing to intrinsic nonlinear elastic forces in pressure-driven flows of polymer solutions [10,11]. The main advantages of viscoelastic microfluidics are the wide usable range of flow rates and lateral migration of particles/cells in a simple straight microchannel [12]. Based on these advantages, viscoelastic non-Newtonian fluids have been used for particle/cell-focusing [11,13,14], size-based particle/cell separation [15,16,17,18,19], and shape-based cell particle/cell separation [20]. More recently, a viscoelastic microfluidic device for a sheathless, continuous concentration of white blood cells (WBCs) was proposed [21]. Using the viscoelastic cell-focusing technique, WBC samples with extremely low concentrations could be concentrated 18-fold with high throughput (350 μL/min).

For further enhancement of the concentration performance of the single-pass operation, a closed-loop operation of the microfluidic channel was introduced [7,22,23]. In a single-pass operation, the concentration performance was determined by the channel design or suction flow control [24]. However, by feeding the concentrated stream of target cells continuously back to the sample inlet through a closed feedback loop, the concentration performance can be improved.

To generate a flow in a microfluidic chip, an external pumping system is required. Conventionally, commercialized pumps, including a syringe pump and a peristaltic pump, have been used, with the advantages of flow stability and wide flow rate range [25,26]. However, these are not suitable for the miniaturization and integration of microfluidic devices owing to their bulky size [27]. The use of piezoelectric pumps can address these limitations, with the advantages of small size, low noise, and low power consumption. Previously, the piezoelectric pump was used for flow actuation in microfluidic channels [27,28,29]. To the best of our knowledge, this is the first study to use piezoelectric pumps for microparticle/cell manipulation.

In this study, we propose a closed-loop viscoelastic microfluidic device to achieve a high concentration ratio of initially rare target cells. In addition, the sheathless viscoelastic concentration technique facilitated the use of parallel channels to improve the device throughput. Miniature and lightweight piezoelectric diaphragm pumps were used for flow actuation in cell concentration applications owing to the reduced flow resistance by using a device with multiple parallel channels. The performance of the piezoelectric pump in the microchannel was evaluated depending on the frequency, applied voltage, channel length, and viscosity of the working fluid. Next, we examined the flow characteristics of 13 μm particles and WBCs to determine the optimal flow conditions for cell concentration. Finally, as examples of clinical utilization of the closed-loop viscoelastic cell concentration system, a lysed blood sample was used to enrich the WBCs for the quality control of the blood transfusion, and the prostate cancer cell line was used for the diagnosis of prostate cancer using urine samples.

## 2. Materials and Methods

### 2.1. Device Fabrication

A polydimethylsiloxane (PDMS) microchannel was fabricated using soft-lithography techniques with a replica mold. The mold was fabricated using an SU-8 negative photoresist (MicroChem, Newton, MA, USA) patterned on a silicon wafer. A 10:1 mixture of the PDMS base and curing agent (Sylgard 184, Dow Corning, Midland, MI, USA) was mixed, cast over the replica mold, degassed in a vacuum chamber, and thermally cured in an oven for 1 h at 80 °C. The cured PDMS channels were peeled off from the mold and bonded on a glass slide with oxygen plasma (CUTE, Femto Science, Hwaseong, Korea).

The high-throughput cell concentration device consisted of four microchannels, each 60 × 125 μm (W × H) with a high aspect ratio (AR = height/width). The device includes a parallel arrangement of channels with a common single inlet and two outlets for each channel. The lengths of the main channel were 1, 2, and 3 cm to determine the optimal length of the main channel after the evaluation of the flow characteristics of cells in the microfluidic device. The width of the expansion region was designed to be 200 μm for monitoring the flow characteristics, and the widths of the outlet trifurcation channels connected to the expansion region were 150, 100, and 150 μm, respectively.

A plastic jig was fabricated by a three-dimensional (3D) printing technique using a 3D printer (ProJet 3510 HD, 3D systems, Rock Hill, SC, USA) to hold a PDMS microchannel device, two commercial 50 mL conical tubes as the sample and waste chamber, two piezoelectric pumps, and a needle valve.

### 2.2. Cell Culture

The prostate cancer cell line DU-145 was obtained from the Korean Cell Line Bank (Seoul, Korea). DU-145 cells were cultured in Falcon T-25 cell culture flasks in 89% RPMI1640 with L-glutamine (300 mg/L), 25 mM HEPES, and 25 mM NaHCO_3_ containing 10% fetal bovine serum and 1% penicillin-streptomycin (all Gibco). Cell culture flasks were kept in a 5% CO_2_-controlled incubator at 37 °C. Cells were prepared for the experiment once 70% confluent by removing the medium and washing the culture flask with 2 mL of 0.05% Trypsin-EDTA (1×, Gibco, Thermo Fisher, Waltham, MA, USA). Next, 4 mL of pre-warmed RPMI was added to neutralize trypsin. After detachment, the cells were spun at 1000 rpm for 3 min and the pellet was used. Cells were passaged every third day.

### 2.3. Sample Preparation

First, 0.1 (*w*/*v*)% hyaluronic acid (HA) sodium salt (357 kDa, Lifecore Biomedical, Chaska, MN, USA) was prepared in phosphate-buffered saline as a viscoelastic non-Newtonian fluid. The high shear viscosity (η∞) and relaxation time (λ) of the solution were measured as 0.89 mPa·s and 0.25 ms, respectively [30]. Fluorescence polystyrene particles 13 μm in diameter were used as an analog to WBCs. The particles were suspended in 0.1% HA solution at approximately 1 × 10^5^ particles/mL.

For sample preparation, single-donor human whole blood and pooled human urine (Innovative Research, Inc., Novi, MI, USA) were used. In brief, by removing the plasma after centrifugation at 800× *g* for 10 min, blood samples with a hematocrit of 70% were prepared to simulate the packed red blood cells for blood transfusion. Thereafter, 1 mL of the prepared blood sample was mixed with 7 mL of 1 × BD FACS lysis buffer (BD Biosciences, Franklin Lakes, NJ, USA), 1 mL of 1 × SYBR Green, and 1 mL of 1 (*w*/*v*)% HA solution, which had a final concentration of 0.1 (*w*/*v*)% HA solution in a total volume of 10 mL. The number of WBCs was counted manually using a hemocytometer. For the final application of the closed-loop viscoelastic concentration system, cultured DU-145 cells were spiked in 9 mL of pooled human urine sample and mixed with 1 mL of 1 (*w*/*v*)% HA solution. The final concentration of DU-145 cells was 1.5 × 10^3^ cells/mL in 0.1 (*w*/*v*)% HA solution.

### 2.4. Experimental Procedure

The injection and suction flow rates were controlled by two piezoelectric micropumps (SDMP320, Takasago, Tokyo, Japan). The piezopump uses a piezodisc to actuate a COC diaphragm and ethylene propylene diene monomer check valves to generate a directed flow [31]. Two piezoelectric micropumps were controlled simultaneously by using a piezoelectric micropump controller (MPC-200B-EU, Takasago), which modulated the frequency and voltage applied to the pump. The input voltage to the controller was 5 VDC, and the output voltage from the controller ranged from 60 to 300 V, which was applied to the piezoelectric pump. Additionally, a micro needle valve (MNV series, Takasago) was used to regulate the flow rate in detail.

To connect the microfluidic channel, two piezopumps and reservoirs, and a Tygon tube with an inner diameter of 0.51 mm were used. The total length of the Tygon tube was approximately 400 mm, which affects the dead volume of the fluidic system.

During the experiment, particles/cells flowing in the microchannel were monitored using an inverted microscope (CKX41, Olympus, Tokyo, Japan) with a high-speed camera (V611, Phantom, St. Louis, MI, USA) and a fluorescent camera (CS230B, Olympus, Tokyo, Japan).

## 3. Results

**Figure 1:**
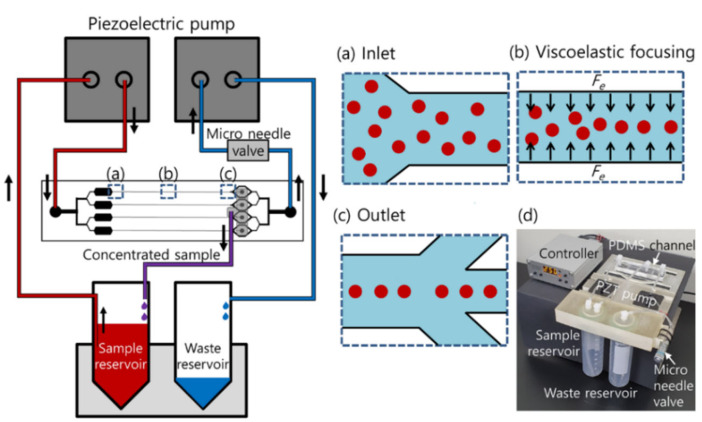
Schematic of the closed-loop system for high-throughput viscoelastic cell concentration. The 3D-printed jig contains a microfluidic device with four parallel channels, two piezoelectric pumps, a sample reservoir, a waste reservoir, and a micro needle valve. (**a**) Cell suspension in a viscoelastic fluid was randomly introduced to the inlet. (**b**) Due to the elastic force, cells were focused in the center of the microchannel. (**c**) At the outlet, tightly focused cells were collected from the center outlet and the additional medium was removed to the side outlets. (**d**) Fabricated closed-loop system for high-throughput viscoelastic cell concentration.

A schematic of the proposed device for high-throughput cell concentration using closed-loop viscoelastic fluid is depicted in Figure 1. As shown in the schematic in Figure 1, the device consisted of four parallel microfluidic channels with a high AR, which has one common inlet and two outlets for each channel. At the inlet, the initial sample containing cells at low concentration was injected into the microchannel as randomly distributed (Figure 1a). In the viscoelastic fluid, the elastic force (*F_e_*) induced by the non-dimensional distribution of the first normal stress difference (*N*_1_) affects the lateral migration of the suspended cells toward the channel center (Figure 1b) [11,32,33]. The channel width was designed to make the blockage ratio (*β = a*/*W*, where *a* is the cell diameter and *W* is the channel width) of the cells higher than 0.1 (*β* ≥ 0.1) to focus the cells at the center plane of the microchannel. Focused cells were collected from the four center outlets connected to a single outlet A (Figure 1c). At outlet B, the suction flow rate was controlled by a piezoelectric pump and a micro needle valve to remove the additional suspending medium and enhance the concentration factor of cells at outlet A. Through the closed-loop system, the collected sample at outlet A was recirculated to the inlet of the microchannel and the cells were finally recovered at a higher concentration factor at outlet A. Figure 1d shows a photograph of the fabricated closed-loop system for high-throughput viscoelastic cell concentration containing two PZT pumps, a controller, two reservoirs, a micro needle valve, and a PDMS channel.

To apply a piezoelectric micropump in the device, the applied frequency- and voltage-dependent flow rates were evaluated. At the microscale, the flow resistance increases drastically because a high surface force becomes predominant over the body force. In the microfluidic flow, the flow rate decreases owing to the pressure drop induced by the flow resistance. The flow resistance of the microchannel can be determined by the channel dimension and solution viscosity, as shown below.

(1) $$R=\frac{128\eta L}{{\pi D_{h}}^{4}}$$

**Figure 2:**
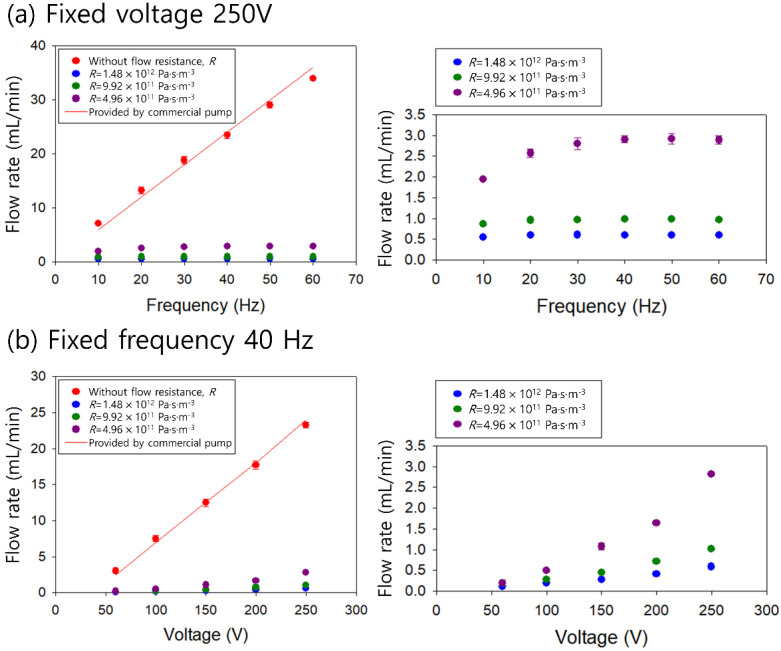
Evaluation of flow rates generated by a piezoelectric pump depending on (**a**) the frequency at the fixed voltage of 250 V and (**b**) the applied voltage at the fixed frequency of 40 Hz without/with 1, 2, and 3 cm-long microchannels with different flow resistances of 4.96 × 10^11^, 9.92 × 10^11^, and 1.48 × 10^12^ Pa·s·m^−3^.

Here, *η*, *L*, and *D_h_* refer to the fluid viscosity, channel length, and hydraulic diameter of the channel, respectively. With the fixed solution viscosity, the flow resistance of the microchannel is proportional to the channel length [34]. Therefore, Figure 2a shows the frequency-dependent flow rates in our system with different flow resistances. The flow resistances were calculated using microchannels with a width of 60 μm, height of 125 μm, and different lengths (1, 2, and 3 cm) as 4.96 × 10^11^, 9.92 × 10^11^, and 1.48 × 10^12^ Pa·s·m^−3^. The flow rate was measured by a flow sensor (SLI, Sensirion, Staefa, Switzerland) with or without a microchannel. Without a microchannel, the piezoelectric pump-induced flow rate increased with the frequency from 7 mL/min at 10 Hz to 34 mL/min at 60 Hz at the macroscale, which was in agreement with the specifications provided by the manufacturers. With the microchannels, the flow rates were much lower than 3 mL/min, owing to the flow resistance of the microchannel. As the channel length increased, the flow rate decreased. With a 3 cm-long channel (*R* = 1.48 × 10^12^ Pa·s·m^−3^), the flow rate increased from 1.9 mL/min at 10 Hz to 2.9 mL/min at 40 Hz. With microchannels of 2 (*R* = 9.92 × 10^11^ Pa·s·m^−3^) and 1 cm lengths (*R* = 4.96 × 10^11^ Pa·s·m^−3^), the flow rate decreased to 0.9 and 0.6 mL/min, respectively. These flow rates were adequate for use in the microfluidic device for viscoelastic concentration.

Figure 2b shows the applied voltage-dependent flow rates at a fixed frequency of 40 Hz. Without flow resistance of the microchannel, the flow rate increased from 3 mL/min at 60 V to 23 mL/min at 250 V. This is in agreement with the specifications of the manufacturer. For all cases, as the applied voltage increased, the flow rate increased. At an applied voltage of 250 V, the flow rates induced in microchannels of 1, 2, and 3 cm were 2.8, 1, and 0.6 mL/min, respectively.

The detailed values of the flow rates and the calculated non-dimensional numbers under the different voltages in the different-length channels are listed in Table 1. Non-dimensional numbers were defined to evaluate the flow characteristics of the particles in the viscoelastic flow. Reynolds number (*Re*) describes the ratio of the inertial force to the viscous force, while the Weissenberg number (*Wi*) describes the ratio of the elastic force to the viscous force.

(2) $$Re=\frac{\rho V_{m}D_{h}}{\eta }$$

(3) $$Wi=\lambda \dot{\gamma_{c}}$$

here, *ρ*, $V_{m}$, and $\dot{\gamma_{c}}$ denote the solution density, mean flow velocity, and characteristic shear rate, respectively. The relative effect of fluid elasticity on inertia can be estimated by the elasticity number (*El*).

(4) $$El=\frac{Wi}{Re}$$

**Figure 3:**
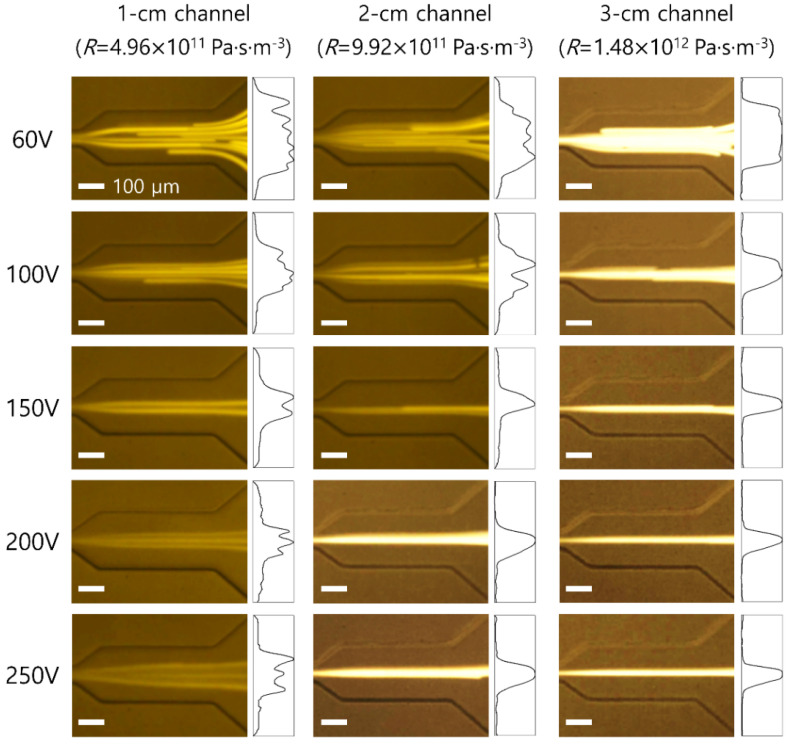
Stacked microscopic images (**left**) and normalized fluorescent intensity in an expansion region (**right**) of 13 μm fluorescent particles at a fixed frequency (40 Hz) with different applied voltages of 60, 100, 150, 200, and 250 V in microchannels with different lengths of 1, 2, and 3 cm, respectively. The scale bar is 100 μm.

To examine the effect of the applied voltages and channel lengths on the flow characteristics of 13 μm particles (blockage ratio *β* = 0.21), particle distributions in the microchannels with lengths of 1, 2, and 3 cm were monitored. Figure 3 shows the stacked microscopic images of flow patterns of 13 μm particles and normalized fluorescence intensities before the outlet trifurcation region of the microchannel. The fluorescence intensity was normalized to the highest intensity value under each flow condition.

In the microchannel with 1 cm length, 13 μm particles were widely distributed near the center region at 60 V. As the applied voltage increased, the flow rate generated by the piezoelectric pump increased, and the distribution of 13 μm particles became narrower near the center as the elastic force increased. At 250 V (*Re* = 142.7, *Wi* = 13.05), 13 μm particles showed three fluorescent streamlines because of the increased inertial effect [35,36]. As the channel length increased, the values of non-dimensional numbers (*Re* and *Wi*) decreased, which resulted in a reduced elastic force. However, owing to the sufficient channel length for particle focusing, 13 μm particles migrated toward the center and focused tighter along the centerline. The lateral position of 13 μm particles was saturated at the center at an applied voltage higher than 150 V in a 2 cm channel and 100 V in a 3 cm channel. To achieve the high-throughput and high-concentration factor in the closed-loop system, the channel length of the four parallel channel devices was selected as 2 cm for tight focusing of cells with minimized flow resistance.

**Figure 4:**
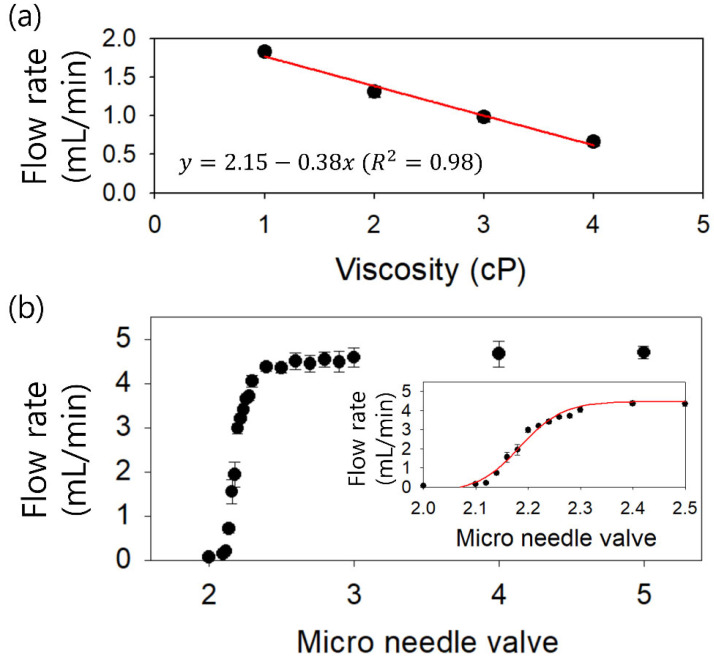
(**a**) Effect of viscosity on the generated flow rate from a PZT pump at a fixed frequency and applied voltage (40 Hz, 250V). (**b**) Micro needle valve-based flow rate control at the fixed frequency and applied voltage (40 Hz, 250 V). An inset graph shows the flow rate using a micro needle valve ranging from 2.0 to 2.5.

For the lysed blood sample, the viscosity was dependent on the hematocrit of the blood sample. The viscosity of lysed blood samples with a normal range of hematocrit (~50%) is ~2.4 cP, while the viscosity of lysed samples using packed red blood cells for blood transfusion (~70% hematocrit) is ~3 cP [21]. Therefore, to confirm the capability of our parallel channel device to utilize the lysed blood samples with various hematocrits, an examination of the viscosity effect on the pumping performance using a piezoelectric pump was required. Figure 4a shows the viscosity-dependent flow rates generated by the piezoelectric pump at a fixed frequency of 40 Hz and a fixed applied voltage of 250 V. Glycerol was diluted in water at various volume ratios to prepare glycerol aqueous solutions with different viscosities of 1, 2, 3, and 4 cP. As the sample viscosity increased, the flow rate decreased because of the increased flow resistance (R∝η, $Q=\frac{∆P}{R}\propto \frac{1}{R}$, ∆*P* is the pressure drop). Using the glycerol solution at 4 cP, the flow rate was measured as ~0.6 mL/min. As the viscosity of the glycerol solution decreased to 3, 2, and 1 cP, the flow rates increased to 1, 1.3, and 1.8 mL/min, respectively. The flow rate is affected by the viscosity of the sample according to the relationship *y* = 2.15 − 0.38*x*. Therefore, even with a high-viscosity sample, for example, lysed sample using 70% hematocrit blood, the throughput of our four parallel channel device could be achieved at up to ~1 mL/min using a piezoelectric pump.

To compose the closed-loop system for a high concentration factor, the flow rate factor was adopted, which is the ratio of the inlet flow rate to the outlet flow rate at the side outlet (outlet B). Two piezoelectric pumps were used, which were modulated simultaneously by a single controller. A micro needle valve was additionally used at outlet B, which enabled more detailed flow rate control with the piezoelectric pump. Figure 4b shows the flow rate reduced by the micro needle valve under the flow generated by the piezoelectric pump at a fixed frequency of 40 Hz and a fixed applied voltage of 250 V. At valve scales higher than 2.3, the micro needle valve was fully open, which had no effect on the flow resistance in the microchannel. The flow rate was saturated at approximately 4.5 mL/min. As the scale of the valve is reduced within 2.3, the flow resistance increases and the flow rate decreases, as shown in the inset of Figure 4b according to the relationship of $y=-0.26+\frac{4.73}{1+e^{\frac{x-2.18}{0.04}}}$ (*R^2^* = 0.98). Therefore, it can be applied to manipulate the suction flow rate at outlet B.

For the application of the closed-loop system for continuous cell concentration, residual WBCs (rWBCs) in the packed red blood cells were used. The diameter of the WBCs was 9–15 μm, which have the blockage ratio of 0.15 ≤ *β ≤* 0.25 [12,30]. The initial volume of the lysed blood sample with a hematocrit of 70% was 10 mL, which was placed in the sample reservoir. Two piezoelectric pumps were operated simultaneously at 250 V and 40 Hz, and the scale of the micro needle valve was set to 2.19, which reduced the suction flow rate at outlet B by approximately 50%. Based on the flow rate measurement in Figure 2, the flow rates for sample injection and suction at outlet B were ~1 and ~0.5 mL/min, respectively, in a 2 cm-long channel.

**Figure 5:**
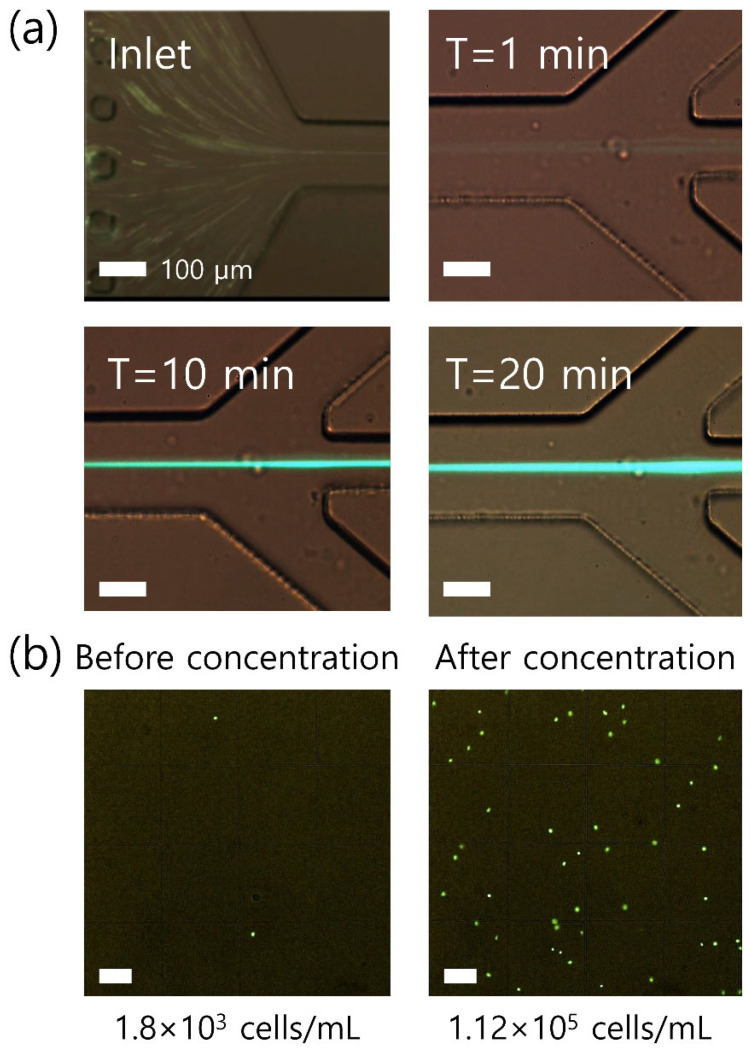
Application of the closed-loop viscoelastic concentration system. (**a**) Time-dependent concentration of WBCs. Randomly injected WBCs in the hematocrit 70% blood sample were tightly focused at the center and concentrated. (**b**) Fluorescent images of the sample before and after the closed-loop concentration process. The scale bar is 100 μm.

Figure 5a shows the time-dependent concentration of rWBCs in the single channel among the four parallel channels in the device. At the inlet, rWBCs were randomly injected into the microchannel. As soon as rWBCs flowed through the microchannel, the cells were focused at the center (T = 1 min in Figure 5a). In the closed-loop system, the focused cells collected at the center outlet were sent back to the inlet through the sample reservoir, while the additional suspending medium was removed from the side outlets by suction. Therefore, the intensity and the width of the fluorescent stream became stronger and thicker, as the closed-loop system was operated for longer (T = 10 and 20 min in Figure 5a). Finally, after 20 min of the closed-loop concentration process, all samples in the sample reservoir were consumed, and the volume of the sample left in the tubing connections as the dead volume was approximately 160 μL.

To evaluate the concentration increase of rWBCs, WBCs in the initial sample and the collected sample after the closed-loop concentration process were examined three times using a fluorescent microscope. The number of cells was counted using a hemocytometer three times, which were $1.78\pm 0.18\times 10^{3}$ cells/mL in the initial sample and $1.13\pm 0.13\times 10^{5}$ cells/mL in the final concentrated sample by 63.4±0.8-fold. Figure 5b shows an example of the sample before and after the closed-loop concentration process.

For a final demonstration, to expand the clinical usability of our closed-loop viscoelastic concentration system, prostate cancer cell line DU-145 cells were used in the urine sample using the same flow conditions in Figure 5. The viscosity of the urine sample was 0.8–1.0 cP at room temperature [37]. The flow rate generated in the microchannel by the piezoelectric pump was higher at the same applied voltage because the viscosity of the urine sample was approximately four times lower than that of the lysed blood sample at 70% hematocrit. According to the relationship *y* = 2.15 − 0.38*x* shown in Figure 4a, the flow rates for sample injection and suction at outlet B were ~1.8 and ~0.9 mL/min, respectively. Therefore, the required sample processing time decreased, that is, ~11 min for a 10 mL urine sample.

**Figure 6:**
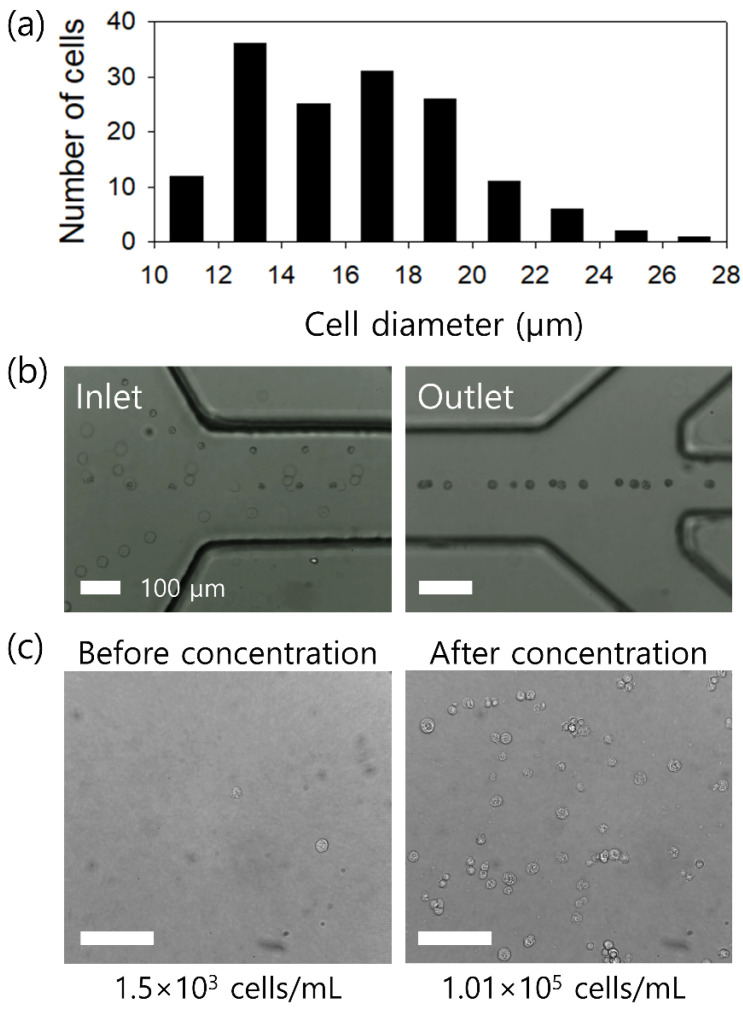
Application of the closed-loop system for the concentration of prostate cancer cells in urine samples. (**a**) Size distribution of DU-145 cells; 16.2 ± 3.4 μm. (**b**) Stacked microscopic images during the concentration process of DU-145 cells. Randomly injected cells were tightly focused at the center in the outlet region. (**c**) Microscopic images of the sample before and after the closed-loop concentration process. The scale bar is 100 μm.

Urine has the powerful advantage of being adopted for liquid biopsy, which is the non-invasive and patient-friendly collection of sufficient volume for analysis without a skilled medical professional. Before use, the size of DU-145 cells was determined, as shown in Figure 6a. The diameter of the DU-145 cells was 16.2 ± 3.4 μm, which has a slightly larger blockage ratio of 0.16 ≤ *β ≤* 0.45 compared to WBCs.

Figure 6b shows the stacked microscopic images at the inlet and outlet of the microchannel during the concentration process of DU-145 cells, which were recorded with a high-speed camera. Due to two-dimensional focusing at the center plane, the image quality of cells at different channel heights could be compromised. At the inlet, DU-145 cells were randomly distributed across the microchannel. When flowing along the microchannel, the cells were affected by the viscoelastic force and tightly focused at the center. After 10 min of running in the closed-loop system, 10-mL of the initial sample in the sample reservoir was consumed and the number of DU-145 cells in the collected sample was enumerated, as shown in Figure 6c. The number of cells was 1.5 × 10^3^ cells/mL in the initial sample and 9.6 × 10^4^ cells/mL in the final concentrated sample, which showed a 64.1-fold increase. Meanwhile, based on the results, the main constituents of urine, including urea, creatinine, sodium, potassium, and chloride, did not interrupt the viscoelasticity effect in the polymer solution.

## 4. Discussion

Using our device, cells can be concentrated without a costly and bulky centrifuge, which also can cause shear-induced cell damages [2,3]. Even cytospin, which is widely used for cell concentration and staining, can cause cells to appear distorted depending on the locations in the smear and the cell concentration [38,39]. In our device, mechanical shear forces induced in macro reservoirs and the tubing connection can be negligible. In addition, cells are focused at the microchannel center, where the shear rate is minimum in the microchannel [40]. Moreover, the most distinctive advantage of microchip-based cell concentration compared to centrifugation is integration with the on-chip post-analysis [41,42,43]. Therefore, our device is suitable for convenient sample preconcentration in clinical settings as benchtop equipment.

Our device can be used universally for cell concentration by modulating the channel width to meet the requirement of blockage ratio (β≥0.1). In the microchannel used in this study, red blood cells, which are known as ~6–8 μm, can be focused at the center of the channel, since the blockage ratio of red blood cells (β=6/60) is higher than 0.1 [44,45]. Meanwhile, cells that cannot be tightly focused at the channel center also can be highly concentrated by modulating the suction flow rate ratio at the outlet [12].

Compared to previous closed-loop microfluidic devices [7,22,23], a bulky pumping instrument such as a peristaltic pump or a syringe pump is not required in our device, which enables benchtop equipment for clinical settings. In addition, due to the advantages of viscoelastic microfluidics [10,11], viscoelastic cell concentration can be achieved in a simple rectangular straight channel without a complex channel design or external force field.

Even though the large volume (10 mL) of the sample was processed in this study, target cells in the small volume can be concentrated in our microfluidic device by integrating further microfluidic interfacing techniques [46,47,48] and micropump systems [49]. Additionally, easy integration with other microfluidic systems, for example, the microfluidic device for cell separation, enables continuous concentration after the separation process. This can be the distinctive characteristic in comparison with the centrifugation that requires a bulk tube-based batch process.

To enhance device performance, the concentration factor can be improved by reducing the dead volume in the tubing connection and fabricating a 3D-printed jig. The use of piezoelectric pumps enables easy integration with other fluidic parts, such as the microfluidic channel and the reservoirs as a fluidic module. Therefore, the closed-loop viscoelastic device provides a compact tool for high-throughput concentration to enhance the detection sensitivity of post-analysis in biomedical and clinical applications. Extremely rare cells, for example, WBCs in cerebrospinal fluid, CD34 (+) cells for stem cell transplantation, and circulating tumor cells (CTCs) for prognosis, can be concentrated and detected at high accuracy and sensitivity [50,51,52,53]. Moreover, the device throughput can be further enhanced by decreasing the flow resistance. Multiple channels in radial arrangement or stacked devices in multiple layers can be adopted to reduce the flow resistance [54,55].

## 5. Conclusions

In summary, we developed a closed-loop continuous concentration system using minimized piezoelectric pumps. To reduce the flow resistance and enhance the device throughput, multiple parallel channels were designed in a single device. The effects of the applied voltage, frequency, and channel length on the piezoelectric pump-induced flow rate were evaluated. Based on the effects of the flow generation conditions, the flow characteristics of 13 μm particles and WBCs were examined. In addition, the effects of the sample viscosity and the use of the micro needle valve were evaluated to verify the device’s applicability. For clinical applications, WBC samples in packed RBC samples (~3 cP) were successfully concentrated with a high concentration ratio (63.4 ± 0.8-fold) within 20 min. In addition, DU-145 prostate cancer cells spiked in urine samples could also be concentrated by ~64.1-fold within ~11 min due to the low viscosity (0.8–1.0 cP) of urine compared to packed RBC blood samples. The proposed closed-loop system is highly applicable to various clinical evaluations, such as WBCs in cerebrospinal fluid, which should be fewer than five, and circulating tumor cells for cancer prognosis.

## Figures and Tables

**Table 1:** Flow rates and the calculated non-dimensional numbers (*Re*, *Wi*, and *El*) at different voltages in the channels with different flow resistances at the fixed frequency of 40 Hz. The unit of the flow rate (*Q*) is mL/min.

Flow Resistance (Pa·s·m^−3^)	60 V	100 V	150 V	200 V	250 V
4.96 × 10^11^	*Q* = 0.20	*Q* = 0.49	*Q* = 1.08	*Q* = 1.64	*Q* = 2.82
	*Re* = 10.12	*Re* = 24.80	*Re* = 54.66	*Re* = 83.00	*Re* = 142.7
	*Wi* = 0.92	*Wi* = 2.26	*Wi* = 5	*Wi* = 7.59	*Wi* = 13.05
	*El* = 0.09	*El* = 0.009	*El* = 0.09	*El* = 0.09	*El* = 0.09
9.92 × 10^11^	*Q* = 0.19	*Q* = 0.27	*Q* = 0.44	*Q* = 0.72	*Q* = 1.01
	*Re* = 9.61	*Re* = 13.66	*Re* = 22.26	*Re* = 36.44	*Re* = 51.11
	*Wi* = 0.87	*Wi* = 1.25	*Wi* = 2.03	*Wi* = 3.33	*Wi* = 4.67
	*El* = 0.09	*El* = 0.09	*El* = 0.09	*El* = 0.09	*El* = 0.09
1.48 × 10^12^	*Q* = 0.11	*Q* = 0.19	*Q* = 0.28	*Q* = 0.42	*Q* = 0.59
	*Re* = 3.18	*Re* = 9.61	*Re* = 14.17	*Re* = 21.25	*Re* = 29.86
	*Wi* = 0.29	*Wi* = 0.87	*Wi* = 1.29	*Wi* = 1.94	*Wi* = 2.73
	*El* = 0.09	*El* = 0.09	*El* = 0.09	*El* = 0.09	*El* = 0.09

## Data Availability

Not applicable.
